# Cognitive health risks posed by social isolation and loneliness in older Korean Americans

**DOI:** 10.1186/s12877-021-02066-4

**Published:** 2021-02-16

**Authors:** Yuri Jang, Eun Young Choi, Nan Sook Park, David A. Chiriboga, Lei Duan, Miyong T. Kim

**Affiliations:** 1 Edward R. Roybal Institute on Aging, Suzanne Dworak-Peck School of Social Work, University of Southern California, 669 West 34th Street, CA 90089-0411 Los Angeles, USA; 2 Suzanne Dworak-Peck School of Social Work, University of Southern California, Los Angeles, USA; 3 Leonard Davis School of Gerontology, University of Southern California, Los Angeles, USA; 4 School of Social Work, University of South Florida, Tampa, USA; 5 Department of Child and Family Studies, University of South Florida, Tampa, USA; 6 School of Nursing, University of Texas at Austin, Austin, USA

**Keywords:** Older Korean Americans, Social isolation, Loneliness, Cognitive impairment

## Abstract

**Background:**

This study examines associations among social isolation, loneliness, and cognitive health risks in older Korean Americans, focusing on the mediating role of loneliness in the relationship between social isolation and objective and subjective measures of cognitive impairment.

**Methods:**

Data are from 2061 participants in the Study of Older Korean Americans, a multi-state survey of Korean immigrants age 60 and older (*M*_age_ = 73.2, *SD* = 7.93). Social isolation was indexed with the Lubben Social Network Scale− 6; loneliness, with the short-form UCLA Loneliness Scale. Objective and subjective measures of cognitive impairment included the Mini-Mental State Examination and a single-item self-rating of cognitive health.

**Results:**

In the logistic regression model for objective cognitive impairment, social isolation was significantly associated, but loneliness was not. In the model for subjective cognitive impairment, both social isolation and loneliness were significant factors. However, the effect of social isolation became non-significant when loneliness was considered, suggesting a potential mediating role of loneliness. The subsequent mediation analysis confirmed that the indirect effect of social isolation on subjective cognitive impairment through loneliness was significant (*B* = .20, *SE* = .03, 95% CI = .12, .28).

**Conclusion:**

Our analyses provide evidence for the proposed mediating effect of loneliness in the relationship between social isolation and subjective cognitive impairment. Intervention efforts should focus on reducing feelings of loneliness experienced by older immigrants, possibly by engaging them in socially meaningful and cognitively stimulating activities.

## Impact statement

We certify that this work is novel in that it differentiated subjective and objective measures of social disconnectedness and cognitive impairment and identified mechanisms underlying them.

## Background

Long recognized as an important social determinant of health, social relationships play an integral role in shaping health and well-being [1]. One line of research on social relationships has focused on the risks to cognitive health posed by social disconnectedness in the later years of life. The findings from systematic reviews of empirical studies on social disconnectedness, however, are inconsistent [2, 3]. Some studies have reported that social disconnectedness leads to poor cognitive function and increased risks of developing dementia [4, 5], whereas other studies have failed to demonstrate statistically significant associations between them [6, 7]. These conflicting findings may be attributable in part to differences among measures, indicating a need to disentangle the contribution of different measures of social disconnectedness and cognitive health and to explore the mechanisms that underlie them.

Social disconnectedness is often assessed with measures of social isolation and loneliness, which are interrelated but distinct concepts. Social isolation represents an objective lack of interpersonal ties or contacts [8]. Loneliness, on the other hand, refers to the subjectively perceived discrepancy between one’s actual and desired social relationships [9]. Given this conceptual difference, it is not surprising that the reported correlation between social isolation and loneliness has been low to moderate [9, 10], with each holding different implications for cognitive health [11, 12].

In addressing the objective and subjective aspects of social disconnectedness, it is plausible to hypothesize that the effect of social isolation on cognitive health may be mediated through the subjective feelings of loneliness. Using a sample of community-dwelling older adults in China, Yang and colleagues [13] provided support for this mediation model by demonstrating that the indirect effect of social isolation on cognitive function through loneliness was significant. Given that their study used a set of binary items to construct a latent variable of social isolation and a single item to represent loneliness, the mediation model needs to be revisited with the employment of psychometrically sound multi-item scales for social isolation and loneliness.

Similar to the construction of social isolation and loneliness, cognitive impairment also requires attention to both objective and subjective indicators. Studies report that the association between objective and subjective measures of cognitive function is low in older populations in general and in racial and ethnic minorities in particular [14–16]. A growing attention has been paid to the discordance between the two measures because subjective rating can be a potential early marker of cognitive impairment and an enabler of help-seeking behaviors [17, 18]. Given their shared but unique natures, objective and subjective indicators of cognitive function may be differentially influenced by social disconnectedness. In particular, previous studies have reported a link between the feelings of loneliness and subjective cognitive impairment, suggesting close connections between these subjective constructs [11, 19]. It seems that older individuals reporting loneliness are more likely to be hypervigilant to perceived external threats such as social isolation and cognitive impairment, which makes them evaluate their cognitive performance in a more negative manner.

Based on the above understanding, in the present study we examine the associations among social isolation, loneliness, and objective and subjective measures of cognitive impairment in older Korean Americans. Koreans represent the fifth largest Asian American subgroup, and the current population of older Korean Americans consists predominantly of foreign-born first-generation immigrants [20]. In Asian cultures, given the fundamental values of collectivism and familism, social networks and support are particularly important [21, 22]. However, as indicated by what has been called the “broken convoy” effect [23], older Asian immigrants often have restricted social networks and limited opportunities to pursue social relationships in their new environments, which makes them susceptible to social isolation and loneliness. In the present study, we examine the cognitive health risks associated with social isolation and loneliness in older Korean Americans, considering both objective and subjective measures of cognitive health. We also explore whether the potential mediating role of loneliness in the relationship between social isolation and cognitive health holds across the objective and subjective measures of cognitive impairment. Covariates were selected based on the literature on social disconnectedness and cognitive health in older adults in general and immigrants in particular, and they include sociodemographic characteristics (age, gender, marital status, education, and perceived financial status), immigration-related characteristics (length of stay in the U.S.), and physical and mental health status (chronic medical conditions and depressive symptoms) [6, 7, 12, 13, 18, 23]. Inclusion of chronic medical conditions and depressive symptoms is in line of the literature demonstrating the interconnectedness among physical, mental, and cognitive health [17, 24].

## Methods

### Participants

Data for the present study are from the Study of Older Korean Americans (SOKA), a multi-state survey of Korean immigrants age 60 and older. The selected states were California, New York, Texas, Hawaii, and Florida, which respectively include 29.3, 8.0, 5.2, 2.7, and 2.2% of the total Korean population resident in the U.S. [25]. In each state, a primary metropolitan statistical area with a representative proportion of Korean Americans was selected: Los Angeles, New York City, Austin, Honolulu, and Tampa. Combined, these sites present a continuum of Korean population densities. The use of multiple sites was intended to address geographic variations and increase generalizability. Community-based samples were recruited by a team of investigators who shared the language and culture of the target population. At each of the five SOKA sites, surveys took place at multiple locations and events (e.g., churches, temples, grocery stores, small group meetings, cultural events) from April 2017 to February 2018. The SOKA questionnaire was in Korean, developed through a back-translation and reconciliation method. Major instruments were selected based on their psychometric qualities in the original and Korean-translated versions. The questionnaire was designed to be self-administered, but trained interviewers were onsite for anyone who needed assistance. Upon completion of the SOKA questionnaire, each participant was also assessed for cognitive function using the Mini-Mental State Examination (MMSE) [26]. Data collection for the project was approved by the Institutional Review Board at the University of Texas at Austin (FWA#00002030), and the procedure involving data collection from human subjects was in accordance with the Declaration of Helsinki. Prior to the survey, written consent was obtained from each participant. A total of 2176 individuals participated in the survey. After removal of those with data missing on the MMSE or subjective cognitive rating or whose cognitive status suggested severe impairment (MMSE score < 10), the final sample for the present study consisted of 2061 participants.

### Measures

#### Social isolation

The Lubben Social Network Scale− 6 (LSNS− 6) [27, 28] was used to indicate social isolation. The scale includes three items on family and a similar set of three items on friends (How many relatives/friends do you see or hear from at least once a month? How many relatives/friends do you feel at ease with such that you can talk with them about private matters? How many relatives/friends do you feel close to such that you could call on them for help?). The respondent answered each question on a 6-point scale (0 = *none* to 5 = *nine or more*), with total scores ranging from 0 to 30. The LSNS-6 has been translated into Korean, and its psychometric properties and cut-off scores have been validated [29, 30]. Internal consistency of the scale in the present sample was high (α = .88). Using the suggested cut-off score [27, 28], participants were identified as either socially connected (LSNS− 6 score ≥ 12) or socially isolated (LSNS− 6 score < 12).

#### Loneliness

Three items were adapted from the short-form UCLA Loneliness Scale [31]. Participants were asked to indicate their responses to the following questions: (1) How often do you feel that you lack companionship? (2) How often do you feel left out? and (3) How often do you feel isolated from others? Each item was rated on a 4-point scale ranging from 1 (*never*) to 4 (*often*). Total scores could range from 3 to 12, with higher scores indicating greater levels of loneliness. The scale has been translated into Korean, and its psychometric properties have been validated [32]. Internal consistency of the scale in the present sample was high (α = .81).

#### Objective cognitive impairment

The MMSE [26] was used as an index of global cognitive function. The MMSE includes items on orientation to time and place, word registration and recall, attention and calculation, language, and visual construction. Responses for each item were scored as 0 (*incorrect*) or 1 (*correct*), and total scores could range from 0 to 30. A score of 24 or below indicates cognitive impairment [26]. The psychometric properties of the Korean version of the MMSE and its cult-off scores have been validated [33, 34]. Internal consistency of the scale was satisfactory (α = .73). In the present analysis, a dichotomized score (MMSE score > 24 = *normal cognition*, MMSE score ≤ 24 = *cognitive impairment*) was used.

#### Subjective cognitive impairment

Participants were asked to rate their overall cognitive health on a 5-point scale, and responses were dichotomized as either positive (0 = *excellent/very good/good*) or negative (1 = *fair/poor*). The latter category was used to indicate subjective cognitive impairment. This single-item rating has been used as a subjective indicator of overall cognitive health, and the dichotomization has been widely accepted [35, 36].

#### Covariates

Sociodemographic variables included age (in years), gender (0 = *male*, 1 = *female*), marital status (0 = *not married*, 1 = *married*), education (0 = ≤*high school graduation,* 1 = > *high school graduation*), and perceived financial status (1 = *below average*, 2 = *average*, 3 = *above average*). Length of stay in the U.S. (in years) was also included as an immigration-related covariate.

As physical and mental health indicators, chronic medical conditions and depressive symptoms were considered. Chronic medical conditions were assessed with a checklist of ten diseases and conditions common in older populations (hypertension, heart disease, stroke, diabetes, cancer, arthritis, liver disease, kidney disease, asthma, and chronic obstructive pulmonary disease), and total count was used in the analysis.

Depressive symptoms were indexed by the Patient Health Questionnaire 2 (PHQ 2), a short form of the PHQ 9 [37]. Participants were asked to indicate how often, over the past 2 weeks, they had been bothered by problems such as “little interest or pleasure in doing things” and “feeling down, depressed or hopeless.” Each item was scored on a 4-point scale ranging from 0 (*not at all*) to 3 (*nearly every day*). Total scores could range from 0 to 6, with higher scores indicating greater levels of depressive symptoms. The scale has been translated into the Korean language, and its psychometric properties have been validated [38]. Internal consistency of the scale in the present sample was high (α = .80).

### Analytical strategy

After reviewing the descriptive characteristics of the sample, bivariate correlations were performed to identify underlying associations among study variables. We also conducted separate logistic regression analyses for objective and subjective measures of cognitive impairment. In each analysis, the direct effect of social isolation was tested, followed by the entry of loneliness. Using the PROCESS macro [39], we examined the hypothesized mediation of loneliness (i.e., the indirect effect of social isolation on cognitive impairments through loneliness). The primary test of an indirect effect was based on the asymmetric distribution of products test using a bootstrapping approach [40], with 95% confidence intervals for the indirect effect estimated using 5000 bootstrap samples. Analyses were conducted after controlling for the effects of covariates (age, gender, marital status, education, perceived financial status, length of stay in the U.S., chronic medical conditions, and depressive symptoms). All analyses were performed using IBM SPSS Statistics 27 (IBM Corp., Armonk, NY).

## Results

### Descriptive characteristics of the sample

Table 1 summarizes the characteristics of the sample. The mean age of the sample was 73.2 years (*SD* = 7.93). About 67% were female, over 60% were married, and 40% had more than 12 years of education. Perceived financial status averaged 1.75 (*SD* = 0.60), and the length of residence in the U.S. averaged 31.4 years (*SD* = 12.1). The average scores for chronic medical conditions and depressive symptoms were 1.57 (*SD* = 1.40) and 1.03 (*SD* = 1.54), respectively. More than 24% of the sample fell in the category of social isolation, and the mean score of loneliness was 4.73 (*SD* = 1.86). The proportions of falling into the categories of objective and subjective cognitive impairment were 18.5 and 33.2%, respectively.

**Table 1:** Descriptive Characteristics of the Sample (*N* = 2061)

Measure	Value
Age, years, *M* ± *SD* (range)	73.2 ± 7.93 (60–100)
Gender (female), %	66.8
Marital status (married), %	60.8
Education (>high school graduation), %	39.7
Perceived financial status, *M* ± *SD* (range)	1.75 ± 0.60 (1–3)
Length of stay in the U.S., years, *M* ± *SD* (range)	31.4 ± 12.1(.17–80)
Chronic medical conditions, *M* ± *SD* (range)	1.57 ± 1.40 (0–10)
Depressive symptoms, *M* ± *SD* (range)	1.03 ± 1.54 (0–6)
Social isolation, %	24.3
Loneliness, *M* ± *SD* (range)	4.73 ± 1.86 (3–12)
Objective cognitive impairment (MMSE ≤24), %	18.5
Subjective cognitive impairment (fair/poor), %	33.2

### Bivariate correlation among study variables

In the bivariate correlations shown in Table 2, all variables were correlated in expected directions and no sign of collinearity was detected. The highest correlation coefficient was between depressive symptoms and loneliness (*r* = .39, *p* < .001), greater symptoms of depression being associated with higher levels of loneliness. Social isolation and loneliness were both associated with unmarried status, lower education and perceived financial status, more chronic medical conditions, and greater levels of depressive symptoms. Advanced age was highly associated with social isolation but not with loneliness. The association between social isolation and loneliness was significant but moderate (*r* = .31, *p* < .001). Both objective and subjective cognitive impairment were associated with advanced age, female gender, unmarried status, lower education and perceived financial status, fewer years of residence in the U.S., more numbers of chronic medical conditions, greater levels of depressive symptoms, being socially isolated, and higher levels of loneliness. A modest association was found between objective and subjective cognitive impairment (*r* = .19, *p* < .001).

**Table 2:** Correlations among Study Variables

	1	2	3	4	5	6	7	8	9	10	11	12
1. Age	–											
2. Female	−.12***	–										
3. Married	−.23***	−.26***	–									
4. >High school graduation	−.08**	−.29***	.16***	–								
5. Perceived financial status	−.18***	−.01	.25***	.24***	–							
6. Length of stay in the U.S.	.17***	−.00	−.02	.13***	.22***	–						
7. Chronic medical conditions	.27***	.11***	−.16***	−.17***	−.22***	.01	–					
8. Depressive symptoms	.10***	.08***	−.17***	−.14***	−.22***	−.03	.20***	–				
9. Social isolation	.09***	−.02	−.15***	−.08***	−.19***	−.03	.05*	.22***	–			
10. Loneliness	.01	−.03	−.11***	−.06**	−.20***	−.03	.13***	.39***	.31***	–		
11. Objective cognitive impairment	.31***	.13***	−.21***	−.26***	−.14***	−.05*	.17***	.12***	.15***	.05*	–	
12. Subjective cognitive impairment	.16***	.09***	−.13***	−.24***	−.27***	−.12***	.22***	.33***	.16***	.28***	.19***	–

*p < .05

**p < .01

***p < .001

### Logistic regression models of objective and subjective cognitive impairment

Table 3 presents the results of the logistic regression models for objective and subjective cognitive impairment. In the model for objective cognitive impairment, social isolation was a significant factor after controlling for the effects of covariates. Being socially isolated increased the odds of objective cognitive impairment by 1.67 times. In the subsequent model, loneliness showed no effect, while social isolation remained significant.

**Table 3:** Multivariate Regression Models of Objective and Subjective Cognitive Impairment

	Odds Ratio (95% Confidence Interval)			
	Objective Cognitive Impairment		Subjective Cognitive Impairment	
**Social Isolation**	1.67 (1.25, 2.24)**	1.70 (1.26, 2.29)**	1.38 (1.07, 1.76)*	1.12 (.86, 1.46)
**Loneliness**	──	.99 (.91, 1.06)	──	1.23 (1.15, 1.31)***
**Covariate**				
Age	1.11 (1.09, 1.13)***	1.11 (1.09, 1.13)***	1.02 (1.01, 1.04)**	1.03 (1.01, 1.05)***
Female	1.64 (1.18, 2.29)**	1.62 (1.15, 2.26)**	1.30 (1.01, 1.68)*	1.40 (1.08, 1.82)*
Married	.66 (.50, .89)**	.66 (.49, .88)**	1.15 (.90, 1.46)	1.19 (.93, 1.51)
> High school graduation	.26 (.18, .37)***	.26 (.18, .37)***	.45 (.36, .58)***	.44 (.35, .57)***
Perceived financial status	1.02 (.79, 1.31)	1.01 (.79, 1.30)	.56 (.45, .69)***	.58 (.47, .71)***
Length of stay in the U.S.	.98 (.97, .99)*	.98 (.97, .99)	.98 (.97, .99)***	.98 (.97, .99)***
Chronic medical conditions	1.08 (.98, 1.18)	1.08 (.98, 1.89)	1.19 (1.09, 1.29)***	1.16 (1.07, 1.26)***
Depressive symptoms	1.02 (.94, 1.10)	1.02 (.94, 1.11)	1.43 (1.33, 1.53)***	1.33 (1.24, 1.44)***
**Summary statistics**				
− 2 Log likelihood	1447.2	1441.6	2014.1	1970.3
χ^2^(df)	376.3 (9)***	374.1 (10)***	414.4 (9)***	455.2 (10)***

*p < .05

**p < .01

***p < .001

In the model for subjective cognitive impairment, the direct effects of both social isolation and loneliness were significant. Social isolation and loneliness were associated with 1.23–1.38 times higher odds of subjective cognitive impairment. However, the initial significance of social isolation disappeared once loneliness was introduced into the model, suggesting a potential mediating effect of loneliness.

Among covariates, advanced age, female gender, lower education, and shorter stay in the U.S. were predictors of both objective and subjective cognitive impairment. Unmarried status was only significant in predicting objective cognitive impairment. On the other hand, lower perceived financial status, more chronic medical conditions, and greater depressive symptoms were significantly associated only with subjective cognitive impairment.

### Mediating effects of loneliness

**Fig. 1:**
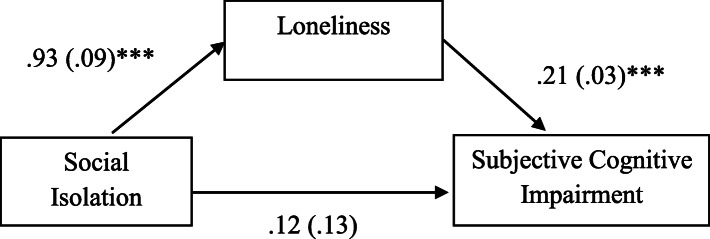
The Mediation Model of Loneliness. *Note.* Numbers indicate unstandardized regression coefficients with standard errors in parentheses. All analyses were conducted controlling for age, gender, marital status, education, length of stay in the U.S., perceived financial status, chronic medical condition, and depressive symptom. Indirect effect of social isolation on subjective cognitive impairment through loneliness = .20 (.03), Bias corrected 95% CI for the indirect effect (.12, .28)

The mediating role of loneliness was further explored using the PROCESS macro. In the model for objective cognitive impairment, the indirect effect of social isolation via loneliness was not significant (*B* = −.01, *SE* = .03), with a 95% bootstrap confidence interval for the indirect effect containing zero (−.09, .06). On the other hand, the indirect effect of social isolation on subjective cognitive impairment was significant (*B* = .20, *SE* = .03), as evidenced by a 95% bootstrap confidence interval for the indirect effect not containing zero (.12, .28). Figure 1 depicts how the effect of social isolation on subjective cognitive impairment was mediated by individuals’ subjective feelings of loneliness.

## Discussion

In this study, we have examined the mechanisms underlying social disconnectedness and cognitive health risks in older Korean Americans, an understudied group in cognitive aging research. Our analyses not only identified the status of social disconnectedness and cognitive health risks in the target population but also found partial evidence for the hypothesized mediation model of loneliness in the relationship between social isolation and cognitive health risks.

More than 24% of the present sample fell into the category of social isolation. This proportion is higher than the 11–20% reported in studies using the same measure (LSNS− 6) with non-Hispanic White older adults [28, 41], but comparable to the 24.7% reported in mixed samples of older Asian Americans [42]. The sample’s observed vulnerability to social isolation provides support for the “broken convoy” effect in the social relations of older immigrants [23]. With regard to cognitive function, nearly 19% of our sample had objectively measured indications of cognitive impairment, but over 33% reported subjective cognitive impairment. These proportions are slightly higher than those in national samples of community-dwelling older adults in the U.S. [35, 36]. But the intercorrelations of both subjective and objective indicators of social disconnectedness (*r* = .31, *p* < .001) and cognitive impairment (*r* = .19, *p* < .001) were only low to moderate, demonstrating individual variations in subjective perceptions and expectations of social relations and cognitive ability [14–16].

The measures of social disconnectedness and cognitive impairment were also positively associated. Although social isolation had similar correlations with both measures of cognitive impairment, the association of loneliness with subjective cognitive impairment (*r* = .28, *p* < .001) was notably stronger than that with objective cognitive impairment (*r* = .05, *p* < .05). In multivariate analyses, social isolation was found to pose a significant risk to both objective and subjective cognitive impairment after controlling for the effects of sociodemographic and immigration-related characteristics, chronic medical conditions, and depressive symptoms. The link between social isolation and objective impairment was unaffected by loneliness. This robust impact of social isolation on objective cognitive impairment reflects the critical role of the structural aspect of social relationships as a potential source of cognitive reserve [2, 3, 43]. On the other hand, loneliness played a critical role in predicting subjective cognitive impairment, and its entry into the analytic model made the effect of social isolation non-significant.

In further analyses, loneliness was found to be a mediator in the association of social isolation with subjective cognitive impairment, but not with objective cognitive impairment. These findings imply that different dimensions of social disconnectedness hold different implications for objective and subjective cognitive health [11, 12, 15, 17]. The findings add to the literature suggesting that social isolation and loneliness may have differential cognitive health consequences and emphasize the need for nuanced assessments. Consequently, these findings provide clinical insights for the care of older immigrants; that is, interventions to reduce feelings of loneliness might be a fruitful strategy for managing or preventing early cognitive decline, possibly by engaging participants in socially meaningful and cognitively stimulating activities.

Some limitations of this study should be noted. Although the SOKA research team made efforts to recruit a diverse, representative group of older Korean Americans in different geographic locations using culturally and linguistically sensitive methods, the study’s non-probability sampling may limit the generalizability of the study findings. The study’s cross-sectional design also restricts causal inferences. The temporal mechanism underlying social disconnectedness and cognitive impairment needs to be further explored using longitudinal data. Future studies should also employ a comprehensive battery of neuropsychological tests, a validated multi-item scale of subjective cognitive impairment, and dementia diagnosis. Also, such variables as employment and engagement in volunteer activities and grandparenting need to be considered since productive aging is closely linked to social connectedness and cognitive health [44].

## Conclusion

Nonetheless, the present study enhances the current understanding of cognitive health among older immigrants by evaluating the contribution of both objective and subjective aspects of social disconnectedness and cognitive impairment. The influence of self-reported loneliness on subjective cognitive impairment suggests that these simple measures may be used as screens in routine health check-ups to detect older immigrants’ social, emotional, and cognitive health risks.

## Data Availability

The dataset used in the study is available from the corresponding author upon request.
